# Supplementary material for: Sequence Analysis and Functional Verification of the Effects of Three Key Structural Genes, PdTHC2’GT, PdCHS and PdCHI, on the Isosalipurposide Synthesis Pathway in Paeonia delavayi var. lutea
Source: Int J Mol Sci. 2022 May 19;23(10):5696. doi: 10.3390/ijms23105696 (PMC9147737; doi:10.3390/ijms23105696)
Supplement: Supplementary file 1 [file ijms-23-05696-s001.zip › Table S1.pdf]

Table S1 Primers used for PCR and qRT-PCR

Primer Name	Forword primer(5' - 3')	Reverse primer(5' - 3')	Description
PdTHC2'GT	ATGACGAAAGCAGAGTTAGTC	TTAGGGCATATTTTGTATGACGTC	PCR
PdCHS	ATGGCTTCAGTTGAAGAAATTCGA	TCACTCACTGATTGTAATTGCAGG	PCR
PdCHI	ATGGGTACTGAACAGGTAATG	TCAATAGTCATTTGTAGATAAC	PCR
PdTHC2'GT	AGCAGACATTTTGGCGTGGC	TGCGCTCCAGTCCATTTGCA	qRT-PCR
PdCHS	ACATTTCGCGGACCTTCGGA	TCAGCACCGACAATAACCGCA	qRT-PCR
PdCHI	AGGAGCTGGCCGAAGATGACAA	TCTCCAGTTGCACGCCATACT	qRT-PCR
NtCHS	TTGGGCTGCAGAGACAAGCT	TTGGGCTGCAGAGACAAGCT	qRT-PCR
NtCHI	CCTGTTGGGGCATTGACGAT	AGTCCTGACACTCTTTCGGCGA	qRT-PCR
PsPP2A	CGTGTTTGGATGTTCTCAAGGC	GGCGAGTGAGTTTTCAGTTGGA	qRT-PCR
NtCP	CACCACAAAGGGCAATCTCA	CCGCCAGTCTTTCGTCTCC	qRT-PCR
