# Supplementary material for: Sequence Analysis and Functional Verification of the Effects of Three Key Structural Genes, PdTHC2’GT, PdCHS and PdCHI, on the Isosalipurposide Synthesis Pathway in Paeonia delavayi var. lutea
Source: Int J Mol Sci. 2022 May 19;23(10):5696. doi: 10.3390/ijms23105696 (PMC9147737; doi:10.3390/ijms23105696)
Supplement: Supplementary file 1 [file ijms-23-05696-s001.zip › Table S2.pdf]

Table S2 Protein sequences used to construct phylogenetic trees

Gene	GenBank Number	Species
CrTHC2'GT	BAF75901.1	*Catharanthus roseus*
CpTHC2'GT	BAF75895.1	*Cyclamen persicum*
DcTHC2'GT	BAF75886.1	*Dianthus caryophyllus*
GmIF7GlcT	NP_001235161.1	*Glycine max*
GeIF7GlcT	BAC78438.1	*Glycyrrhiza echinata*
Mt7GT	AAW56091	*Medicago truncatula*
Lb7GlcT	BAD06874	*Lycium barbarum*
SbF7GlcT	BAA83484.1	*Scutellaria baicalensis*
Ih5GT	BAD06874	*Iris × hollandica*
PhA5GlcT	BAA89009.1	*Petunia × hybrida*
ThA5GlcT	BAC54093	*Torenia hybrida*
Per5GT	BAA36421	*Perilla frutescens* var. *crispa*
GhA5GlcT	Q9ZR25.1	*Glandularia × hybrida*
Gh5GT	BAA36423	*Glandularia × hybrida*
Dc3GT1	BAD52003.1	*Dianthus caryophyllus*
VmF3GalT	BAA36972.1	*Vigna mungo*
Ih3GT	BAD83701.1	*Iris × hollandica*
Hg3GT	P14726	*Hordeum vulgare*
ZmF3GT	P16165.1	*Zea mays*
