# Supplementary material for: Sequence Analysis and Functional Verification of the Effects of Three Key Structural Genes, PdTHC2’GT, PdCHS and PdCHI, on the Isosalipurposide Synthesis Pathway in Paeonia delavayi var. lutea
Source: Int J Mol Sci. 2022 May 19;23(10):5696. doi: 10.3390/ijms23105696 (PMC9147737; doi:10.3390/ijms23105696)
Supplement: Supplementary file 1 [file ijms-23-05696-s001.zip › Table S3.pdf]

Table S3 Primers used for fusing GFP and pCAMBIA1302

Gene	primer(5' - 3')	Restriction Enzyme cutting site	Description
PdCHS-F	tctctctcaagcttggatccATGGCTTCAGTTGAAGAAAT	BamHI	Subcellular localization
PdCHS-R	cgggtcatgagctcctgcagCTCACTGATTGTAATTGCAG	PstI	Subcellular localization
PdTHC2'GT-F	tctctctcaagcttggatccATGACGAAAGCAGAGTTAGTC	BamHI	Subcellular localization
PdTHC2'GT-R	cgggtcatgagctcctgcagGGGCATATTTTGTATGACGTC	PstI	Subcellular localization
PdCHI-F	tctctctcaagcttggatccATGGGTACTGAACAGGTAAT	BamHI	Subcellular localization
PdCHI-R	cgggtcatgagctcctgcagATAGTCATTTGTAGATAACT	PstI	Subcellular localization
PdCHS-F	gaacacgggggactcttgacATGGCTTCAGTTGAAGAAAT	NcoI	stable transformation
PdCHS-R	aaaagttcttctcctttactagCTCACTGATTGTAATTGCAG	SpeI	stable transformation
PdTHC2'GT-F	gaacacgggggactcttgacATGACGAAAGCAGAGTTAGTC	NcoI	stable transformation
PdTHC2'GT-R	aaaagttcttctcctttactagGGGCATATTTTGTATGACGTC	SpeI	stable transformation
PdCHI-F	gaacacgggggactcttgacATGGGTACTGAACAGGTAAT	NcoI	stable transformation
PdCHI-R	aaaagttcttctcctttactagATAGTCATTTGTAGATAACT	SpeI	stable transformation
